# Trends in Opioid Prescriptions Based on the Medical Information Mart for Intensive Care IV (MIMIC-IV) Database From 2008 to 2022

**DOI:** 10.7759/cureus.116382

**Published:** 2026-09-16

**Authors:** Zachary Negin, Kyra Cui, Amar Gupta

**Affiliations:** 1 Engineering, Washington Township High School, Sewell, USA; 2 Clinical Support Decision Group, Massachusetts Institute of Technology, Cambridge, USA

**Keywords:** mimic-iv database, opioid epidemic, pain management, prescriptions, trends

## Abstract

Background: Opioid prescription practices in the United States have changed substantially over the past 25 years in response to government policies, medical guidelines, and the opioid epidemic.

Methodology: The Medical Information Mart for Intensive Care IV (MIMIC-IV) version 3.1 database comprises de-identified patient encounters from Beth Israel Deaconess Medical Center (BIDMC) in Massachusetts, United States. Data were analyzed using Structured Query Language (SQL) via Google BigQuery. Patients were grouped into three-year cohorts to further protect confidentiality.

Results: Between 2008 and 2022, data from 364,627 patient admissions were evaluated, including 1,150,944 opioid prescriptions. The percentage of patients receiving opioids decreased from 46.5% (47,273 of 101,607) in the 2008-2010 cohort to 33.6% (16,545 of 49,173) in the 2020-2022 cohort. The number of prescriptions per patient also decreased, from 9.9 in the 2008-2010 cohort to 5.8 in the 2020-2022 cohort. Furthermore, the total prescribed strength per patient decreased from 829 morphine milligram equivalents (MME) in the 2008-2010 cohort to 508 MME in the 2020-2022 cohort. These decreases were consistent across all cohorts during the study period.

Conclusions: These findings are consistent with other studies from the United States and internationally demonstrating declines in the volume and potency of opioid prescriptions during this period. Despite these reductions, opioid-related deaths increased over the same period. These trends persisted across the recognized waves of the opioid epidemic. Although previous studies have reported opioid prescription data from the MIMIC database, our findings provide the most up-to-date analysis spanning the opioid epidemic.

## Introduction

Poppy extracts have been used to treat pain for thousands of years. Although some people have always been harmed by these medicines, many more have obtained relief from suffering. The American opioid epidemic has claimed hundreds of thousands of lives over the past three decades through addiction, overdose, and, more recently, fentanyl poisoning [1]. The Centers for Disease Control and Prevention (CDC) and other groups have divided the opioid epidemic into four overlapping phases [2]:

Wave 1 (late 1990s through 2010): The first phase of the epidemic was driven by intensified prescribing of semi-synthetic opioids such as OxyContin. From 1999 to 2010, the CDC found that rates of prescription opioid sales quadrupled. This increase was accompanied by a doubling of opioid-related overdose deaths.

Wave 2 (2010-2013): Characterized by efforts to tighten opioid prescribing, this period saw a spike in heroin-related addiction and overdose. Declining prices, caused by a shift in manufacturing from South America to Mexico, made this illicit narcotic more accessible. Under these conditions, heroin surpassed many popular prescription formulations as the deadliest opioid.

Wave 3 (2013 to the present): Fully synthetic opioids, primarily fentanyl, entered the market. Many overdose deaths resulted from illicit, non-pharmaceutical fentanyl. These opioids often have higher potency than their natural counterparts, increasing the risk of overdose and addiction. The third wave of the opioid epidemic saw another doubling of opioid-related overdose deaths.

Wave 4 (late 2010s onward): Now described in newer literature, this wave is marked by a rise in polysubstance-related deaths. In particular, the combination of fentanyl with stimulants such as cocaine or methamphetamine has become concerningly common in illicit drug markets.

These waves never cleanly replaced one another [1]. Prescription-opioid and heroin-related deaths continued as fentanyl use rose. The unpredictable course of the epidemic reflects the uneven evolution of policies intended to address both prescription and illicit opioid addiction. Our study aims to relate hospital data to this timeline.

## Materials and methods

Study population

This retrospective analysis evaluated prescription orders for hospitalized patients older than 18 years who were admitted from 2008 to 2022. The Medical Information Mart for Intensive Care IV (MIMIC-IV) v3.1 was used for data analysis [3,4]. This is the most recent release of the large dataset from Beth Israel Deaconess Medical Center, a major academic medical center in Boston, Massachusetts. The data include emergency department visits, hospital admissions, and intensive care unit admissions. The most recent dataset covers patients from 2008 to 2022 and comprises 364,627 unique patients among 546,028 hospitalizations and 94,458 hospital admissions.

Data management

Patient information was de-identified to protect confidentiality. Best practices, including training through the Collaborative Institutional Training Initiative (CITI) for Data or Specimens Only Research, were followed to ensure data security.

Dates of admission are deliberately obscured to further protect patients. By linking patient data to 3-year intervals, referred to as anchor years, the database still permits date-based analysis. These anchor-year intervals are 2008-2010, 2011-2013, 2014-2016, 2017-2019, and 2020-2022.

After authorization was obtained from PhysioNet.org, the data were imported into Google BigQuery for extraction. Data were extracted from the following MIMIC-IV subsets: mimiciv_3_1_hosp.prescriptions and mimiciv_3_1_hosp.patients. SQL queries were then used to analyze the data.

## Results

Percentage of patients receiving opioids

The proportion of patients who received opioids decreased over the study period (Table 1). In the first anchor-year cohort, 46.5% (47,273 of 101,607) of patients at Beth Israel Deaconess Medical Center (BIDMC) received at least one opioid prescription. This percentage declined in each successive three-year interval. A decade later, in the 2017-2019 cohort, the percentage of patients had fallen to 34.4% (22,651 of 65,941), a 26% relative decrease.

**Table 1:** Overall unique patients and percent receiving at least one opioid prescription.

Real-year group	Unique patients	Unique patients who received opioids	Percent receiving opioids
2008-2010	101,607	47,273	46.5%
2011-2013	76,266	31,377	41.1%
2014-2016	71,640	27,804	38.8%
2017-2019	65,941	22,651	34.4%
2020-2022	49,173	16,545	33.6%

Amount of opioids received

The analysis examined the amount of opioids patients received in two ways. The database reported both the number of prescriptions per patient and the total quantity of opioids administered. From 2008 to 2022, the number of prescriptions per patient was nearly halved, from 9.9 to 5.8. This part of the analysis excluded patients who received no opioid prescriptions.

Further analysis of the same patients showed that those who received opioids were given less medication overall. In the peak 2008-2010 cohort, patients who received opioids were prescribed 829 morphine milligram equivalents (MME). This amount declined substantially over time; in the final 2020-2022 anchor-year cohort, patients received an average of only 508 MME (Table 2).

**Table 2:** Average opioid prescriptions and total morphine milligram equivalents per patient.

Real-year group	Total opioid prescriptions	Unique patients who received opioids	Number of prescriptions per patient	Average morphine milligram equivalents per patient
2008-2010	469,283	47,273	9.9	829
2011-2013	239,526	31,377	7.7	678
2014-2016	199,479	27,804	7.2	690
2017-2019	146,451	22,651	6.5	552
2020-2022	96,205	16,545	5.8	508

**Figure 1:**
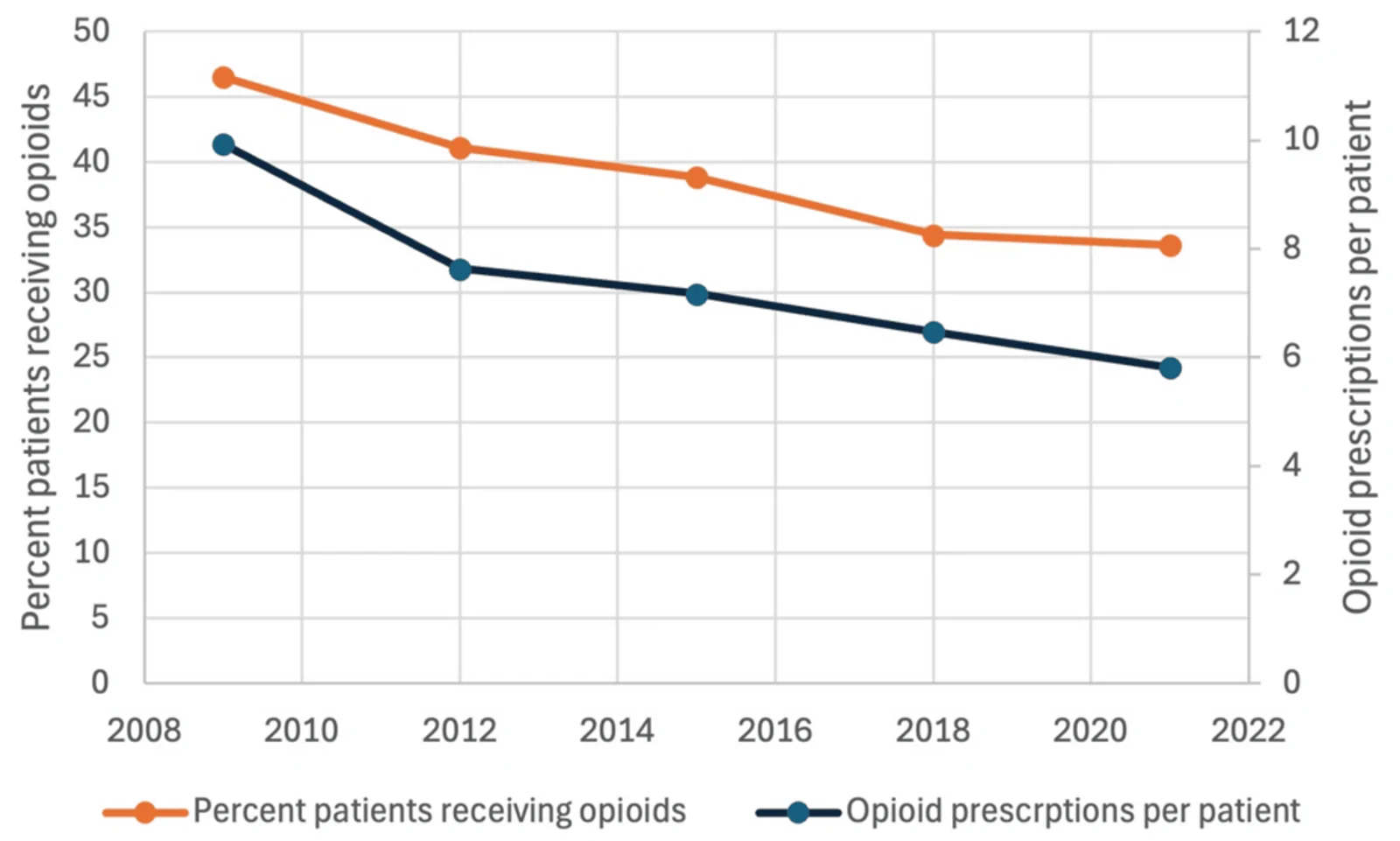
Trend in the percentage of patients receiving opioids and the number of opioid prescriptions per patient.

The data show a decrease in the amount of opioids prescribed, reflecting both a lower percentage of patients receiving opioids and fewer prescriptions per patient among those who received them (Figure 1).

Data explanation/limitations

A note on the data is that the overall number of patients declined over the analysis period. This is an artifact of the dataset and does not represent a decrease in patient volume. MIMIC-IV assigns each unique patient to an anchor-year group based on the first admission. As a result, all patients in the first group were counted as unique, while patients who returned in later anchor-year groups remained assigned to the earlier group. 

Opioid-dosing data in MIMIC-IV have significant limitations. We included the following drugs in our search parameters: morphine, fentanyl, hydromorphone, oxycodone, hydrocodone, codeine, tramadol, and methadone. The number of prescriptions is relatively straightforward. The database is not fully consistent with as-needed (PRN) dosing or with MME conversions between intravenous (IV) and oral (PO) administration. For example, if a patient was given IV morphine as opposed to PO morphine, this would constitute a higher MME, which is not reflected in the data. To this extent, we included MME dosing as an exploratory analysis. The percentage of patients receiving opioids, as well as the number of prescriptions, are very robust data points.

## Discussion

In this analysis, we present the most recent update of opioid prescription trends based on the MIMIC-IV database from BIDMC, a major academic hospital in Boston, Massachusetts. The scale of mortality from the opioid epidemic has had a substantial impact on government, pharmaceutical, and medical society policies.

We believe this analysis is the first published study of MIMIC-IV v3.1 for this specific question. Prior work, such as Chandra et al., evaluated earlier data, including MIMIC-III and earlier versions of MIMIC-IV [5]. Numerous other studies have also examined opioid prescription trends across different procedures, time periods, conditions, and countries.

Our dataset begins in 2008, marking the start of the second wave of the opioid epidemic, when use shifted away from prescription opioids toward street drugs. It then spans the third wave, dominated by fentanyl from 2013 to 2019, and ends at the beginning of the fourth wave, characterized by the rise of synthetic polysubstance combinations [1,6].

Transition from wave 1 to wave 2 of the epidemic

Trends in hospital opioid use were robust and consistent across all measures. Table 1 shows that the proportion of patients who received opioids declined steadily and substantially throughout the study period. Table 2 shows that even among patients who received opioid pain management, the number of prescriptions and overall dosing, standardized to MME, also declined. The shift from wave 1 to wave 2 was driven mainly by changes in the regulation of prescription opioids, particularly controversy surrounding OxyContin and federal and state efforts to police and curb prescribing of these powerful pharmaceuticals [7]. Prescription-monitoring programs enabled data analytics so that physicians could track patients, pharmaceutical companies could track pharmacies, and licensing agencies could monitor prescribers [8]. This pattern aligns with our data, which show the largest drop in prescriptions between the 2008-2010 and 2011-2013 cohorts.

Similar findings have been reported by other researchers examining the effects of policy changes on prescribing by surgeons, inpatient providers, and the general population [9-13]. There is also evidence that stricter government monitoring and enforcement led many pain-management clinics to close [14]. Although these policies helped stop fraud and abuse, patients who were addicted to prescribed opioids were left without medically supervised treatment. Thus, the transition from wave 1 to wave 2 coincided with a large increase in opioid-related deaths.

Transition between wave 2 and wave 3 of the epidemic

Despite falling opioid prescription rates, opioid-related morbidity and mortality continued to rise. As awareness grew that these prescriptions can lead to opioid use disorder and overdose, prescribing continued to decline [15]. Physicians not only wrote fewer prescriptions but also provided them for shorter durations [16]. As our analysis shows, both the number and potency of prescriptions continued to fall through this period.

Even so, deaths continued to increase despite tighter policing and guidelines. One reason is the delay between initiation of pain medication and overdose death, which averages 5.2 years [16]. In addition, the rising potency of street drugs - specifically, the shift from heroin to fentanyl - led to more overdoses and poisonings [17].

Wave 3 of the epidemic and beyond

By wave 3, overprescribing of opioids during wave 1 still accounted for a large share of morbidity and mortality because so many years had elapsed.

Studies of U.S. hospital systems are consistent with our more recent data. Opioid prescriptions continued to fall. The rate of decrease was relatively stable over time as further restrictions and government policies were implemented [18-20]. Opioid prescription rates and quantities also varied substantially by demographic and other characteristics. Other investigators found that although prescriptions decreased significantly in all populations, disparities by race and other demographics persisted [5]. Innovations in pain management, such as postoperative guidelines, have also helped limit opioid prescriptions [21,22].

**Figure 2:**
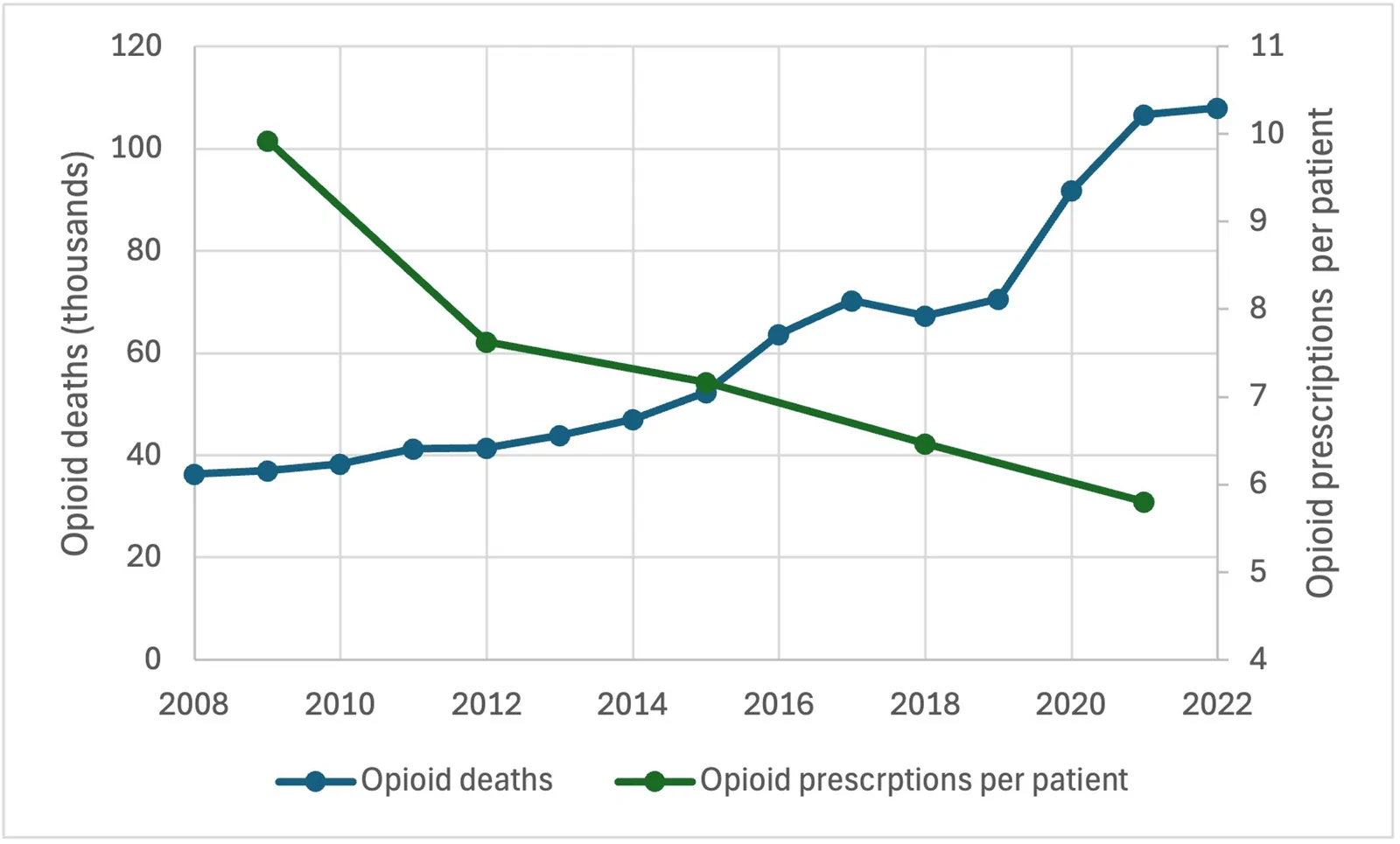
Comparison of opioid deaths and average opioid prescriptions per patient.

**Figure 3:**
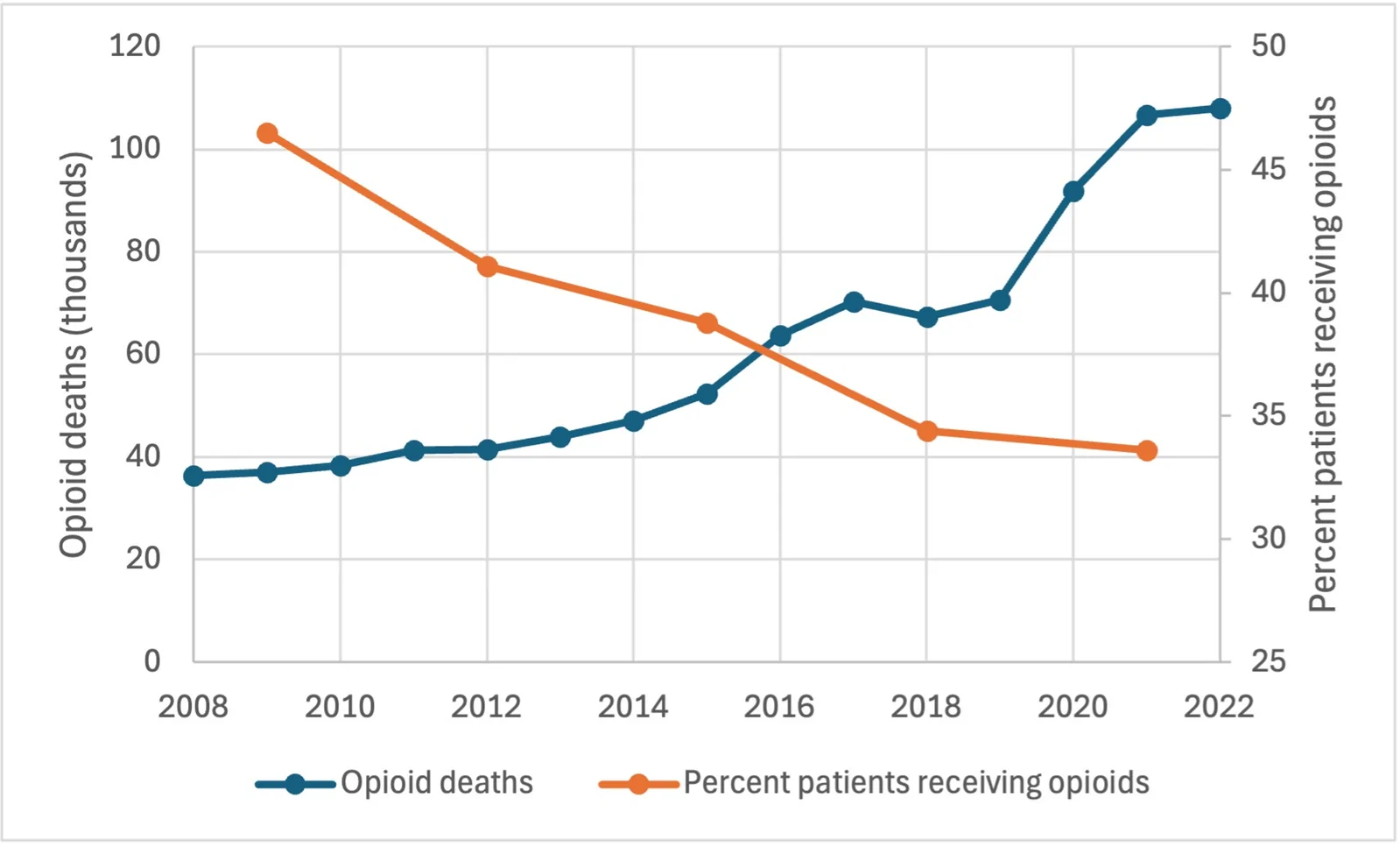
Comparison of opioid deaths and percentage of patients receiving opioids.

Fewer analyses have used more recent databases for this question. Our data extend past 2020 into what is beginning to be called wave 4. We found that declining opioid administration and rising death rates both continued more than a decade after the epidemic began. By 2020, the harms of opioid prescribing were already widely recognized in the medical community, and policies had been in place for years. Even so, the rate and potency of prescriptions continued to fall. Despite that continued decline in narcotic prescribing, mortality has continued to rise (Figures 2-3).

International studies

Several papers from countries outside the United States have examined global efforts to reduce opioid use disorder and fatal opioid overdoses.

To address the limited research on opioid prescription trends in Canada, Rebić et al. [23] analyzed pharmaceutical opioid dispensing data from six provinces between January 1, 2018, and December 31, 2022. During that period, the prevalence of opioid prescribing declined by 11.1%, and the annual incidence fell by 7.9%. There was substantial variation among provinces, with Manitoba having the highest prevalence of opioid use and British Columbia the lowest. Opioid use rates were higher among women, people in low-income areas, and older adults.

In a study of 4,273 patients from 144 hospitals in 25 countries, the TASMAN Collaborative [24] sought to identify factors associated with opioid consumption after common surgical procedures. In the first seven days after discharge, a mean of 179 oral morphine milligram equivalents was prescribed, but patients consumed an average of only 79. Higher prescribed doses were associated with greater opioid consumption during follow-up. Overall, patients prescribed more than 100 oral morphine milligram equivalents were at increased risk of being given more opioids than needed.

The opioid epidemic is a global but uneven problem [25]. Studies of trends outside the United States help place U.S. prescribing practices and their consequences in an international context.

Limitations and future directions

Our results showed a significant decline both in the percentage of patients receiving opioid prescriptions and in the amount prescribed. These findings parallel trends reported in other states and countries.

Although restriction to a single medical center provided internal consistency, the data represent only one hospital in one state. Future studies could examine larger datasets that include more health systems. Second, as noted above, MME calculations were limited by the available data, which lacked clear information on as-needed dosing. More recent versions of the database include these details, but they cannot be compared directly with earlier years, which makes trend analysis difficult. As such, the MME data are presented only as an exploratory analysis. This limitation does not affect the percentage of patients receiving prescriptions or the number of prescriptions per patient, which are more robust measures.

## Conclusions

The decrease in prescriptions demonstrates a clear and continued decrease over the entire study period. During the same period, the number of opioid-related deaths was also noted in the United States. Although prescription opioids such as OxyContin are potentially addictive and harmful, their purity and dose were known. After the epidemic shifted to street drugs, mortality trended upward. With the introduction of fentanyl in the mid-2010s, deaths rose sharply again as fentanyl poisoning compounded the dangers of illicit drugs. Our findings were consistent with this pattern: U.S. overdose deaths were plotted against the percentage of patients receiving opioid prescriptions and the number of prescriptions per patient. This 24-year span of data from more than 350,000 patients is robust and represents the most up-to-date analysis of the MIMIC system.
